# Quantitative Analysis of Alcohol, Sugar, and Tartaric Acid in Alcoholic Beverages Using Attenuated Total Reflectance Spectroscopy

**DOI:** 10.1155/JAMMC/2006/45102

**Published:** 2006-05-31

**Authors:** R. Nagarajan, A. Gupta, R. Mehrotra, M. M. Bajaj

**Affiliations:** 1 Optical Radiation Standards National Physical Laboratory K. S. Krishnan Road New Delhi 110012 India; 2 Department of Chemistry Dyal Singh College University of Delhi Lodi Road New Delhi 110 003 India; 3 Department of Physics and Astrophysics University of Delhi Delhi 110 007 India

## Abstract

Mid-infrared (MIR) spectroscopy in attenuated total reflectance (ATR) mode was used for quantifying ethanol, sucrose, and tartaric acid in alcoholic beverages. One hundred synthetic samples were prepared with different ethanol, sucrose, and tartaric acid concentrations. Experiments were carried out on Bio-Rad 175 C FTS using an ATR accessory. Spectra were recorded in the wavelength region 600–4000 ${\text{cm}}^{-1}$. Calibration was performed using partial least squares (PLS) algorithm. Commercially available alcoholic beverages (gin, rum, vodka, etc.) were experimented and concentration of ethanol in these samples was predicted using the developed calibration model. Chemical analysis of these commercial samples was carried out in order to compare the results. The agreement between ATR results with those of chemical analysis revealed good reliability and repeatability of the technique used.


Hindawi Publishing Corporation
Journal of Automated Methods and Management in Chemistry
Volume 2006, Article ID 45102, Pages 1–5
DOI 10.1155/JAMMC/2006/45102

### Quantitative Analysis of Alcohol, Sugar, and Tartaric Acid in Alcoholic Beverages Using Attenuated Total Reflectance Spectroscopy

**R. Nagarajan,[1] A. Gupta,[2] R. Mehrotra,[1] and M. M. Bajaj[3]**

[1] *Optical Radiation Standards, National Physical Laboratory, K. S. Krishnan Road, New Delhi 110012, India*
[2] *Department of Chemistry, Dyal Singh College, University of Delhi, Lodi Road, New Delhi 110 003, India*
[3] *Department of Physics and Astrophysics, University of Delhi, Delhi 110 007, India*

Received 26 October 2005; Revised 19 December 2005; Accepted 18 January 2006

Mid-infrared (MIR) spectroscopy in attenuated total reflectance (ATR) mode was used for quantifying ethanol, sucrose, and tartaric acid in alcoholic beverages. One hundred synthetic samples were prepared with different ethanol, sucrose, and tartaric acid concentrations. Experiments were carried out on Bio-Rad 175 C FTS using an ATR accessory. Spectra were recorded in the wavelength region 600–4000 $cm^{-1}$. Calibration was performed using partial least squares (PLS) algorithm. Commercially available alcoholic beverages (gin, rum, vodka, etc.) were experimented and concentration of ethanol in these samples was predicted using the developed calibration model. Chemical analysis of these commercial samples was carried out in order to compare the results. The agreement between ATR results with those of chemical analysis revealed good reliability and repeatability of the technique used.

Copyright © 2006 R. Nagarajan et al. This is an open access article distributed under the Creative Commons Attribution License, which permits unrestricted use, distribution, and reproduction in any medium, provided the original work is properly cited.

### 1. INTRODUCTION

Brewing industry has ever-increasing demand for quality products like wine and distilled spirits. The products should satisfy quality control parameters before commercialization. To achieve this, industry needs an efficient technique to monitor the quality parameters that are effective in controlling the production process. Different methods exist for understanding and characterizing the quality of beverages or to quantify ethanol to meet customer's demands [1–5]. Enzymatic determination of ethanol, for example, has undergone considerable development and a series of enzymatic methods based on different techniques is available in literature [6–8].

Most of the wet chemical methods are toxic and involve hazardous chemicals in addition to being time consuming. Hence, various laboratory techniques have been refined at times to achieve the goal. Infrared spectroscopy based methods for analysis of wine and determination of ethanol in other alcoholic beverages are recently emerging because of their versatility, efficiency, being cost effective, fast and noninvasiveness [9–14].

A number of researchers tried to exploit mid-infrared transmission spectroscopic studies in brewing industry [15–17]. They observed strong absorption of water in mid-infrared region that posed problems in spectral analysis. As a result, high prediction errors were reported. The use of attenuated total reflectance technique is shown to be a far better choice for analyzing biological samples. The focus of this article is to develop a calibration method using an ATR technique for quantification of ethanol, sugar, and tartaric acid in alcoholic beverages. The calibration model was built on synthetic samples using partial least squares algorithm.

### 2. EXPERIMENTAL DETAILS

Absolute ethyl alcohol (99.8 percent), sucrose (99.7 percent), and tartaric acid (99.7 percent) were procured from market for sample preparation. Considering the concentration of constituents in commercially available alcoholic beverages, hundred synthetic samples of different concentrations were prepared for calibration in the lab with the specifications: ethanol (10–50 percent), sucrose (0.8–8.8 percent), and tartaric acid (0.5–8.2 percent). The samples were prepared just prior to collecting the spectra. Six commercially available alcoholic beverages branded Brihans Brandy, Red Riband Vodka, Blue Riband Ginn, Old-Monk Rum, McDowell's Whisky, and Royal Visa Red wine were procured from market for predicting ethanol concentration.

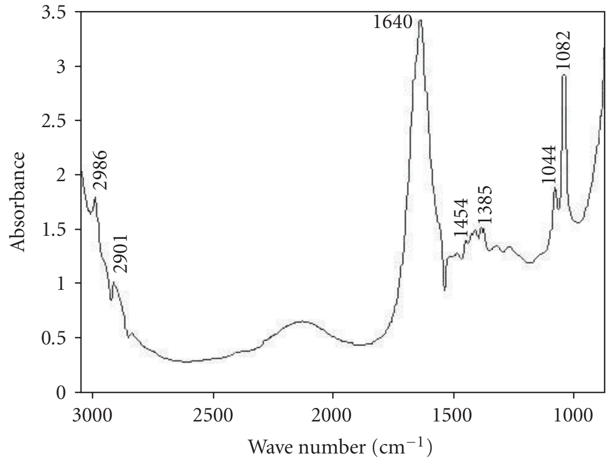

Figure 1: ATR spectrum of synthetic sample in 700–3200 $cm^{-1}$ region.

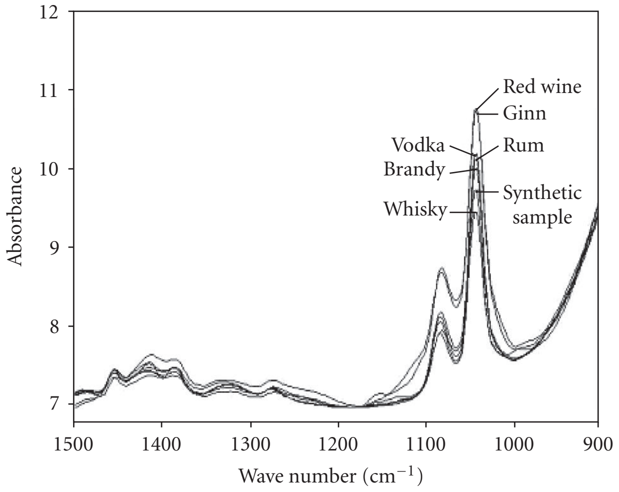

Figure 2: Overlaid ATR spectra of synthetic and real samples in 900–1500 $cm^{-1}$ region.

ATR spectra of all samples were collected on Bio-Rad 175 C FTS spectrophotometer with a resolution of 4 $cm^{-1}$ in the wavelength region 600–4000 $cm^{-1}$. 64 scans were collected for every spectral acquisition using air as background. Horizontal-type ATR accessory housing zinc selenide (ZnSe) crystal (reflection occurs at 45°) was used for spectral measurements. The spectral data were collected for all the 100 synthetic samples as well as the above mentioned six real samples.

Alcohol content in all the above said six real samples was determined chemically as per prescribed Indian standards [18]. 50 ml of alcoholic drink was taken in a round-bottom flask and was subjected to distillation. The distillate (alcohol) was collected at 70°C. The distillate was taken in a 50 ml volumetric flask and the volume was made up. The specific gravity of the diluted distillate was calculated using a specific gravity tube. The ethanol concentration was calculated using the tables quoted in standards.

### 3. RESULTS AND DISCUSSION

Figure 1 depicts the representative spectrum of the synthetic sample. The C−H stretch absorption bands at 2986 $cm^{-1}$ and 2901 $cm^{-1}$ may be attributed to ethanol and tartaric acid present in the sample. An intense sharp band vivid at 1640 $cm^{-1}$ may be due to C=O stretch because of tartaric acid. The three C−O stretch absorption bands at 1385 $cm^{-1}$, 1044 $cm^{-1}$, and 1082 $cm^{-1}$ may be due to tartaric acid, ethyl alcohol, and sucrose, respectively. All the peaks described above are already defined in the literature [19]. Wavelength selection is important for any calibration to be accurate. Hence, the spectral region 900–1500 $cm^{-1}$ was chosen for calibration to carry out this analysis since the region houses the major absorption bands of all the three components undertaken for this study.

The overlaid spectra of synthetic and real samples are shown in Figure 2. The position matching of absorption peaks of real samples with synthetic samples in Figure 2 ensures the substitution of synthetic samples for the real ones, to develop the calibration model. All the hundred synthetic samples were used for calibration. All the spectra accounted for calibration were mean centered. 312 data points were used for calibration.

Cross validation, a method that removes one sample at a time from the calibration set and uses it for prediction, was performed. The optimum number of the principal components (PC), which minimized the sum of the residuals, is given below,

$$\text{PRESS} = \sum_{i=1}^{m} \left(Y_{\text{MIR}} - Y_{\text{REF}}\right)^2, \quad (1)$$

where $m$ is the number of samples used to constitute the calibration model, $Y_{\text{REF}}$ is the reference value, and $Y_{\text{MIR}}$ is the value provided by the model. Table 1 summarizes the statistical data for all the components analyzed.

The score plot of PC1 versus PC2 for ethanol, sucrose, and tartaric acid is shown in Figures 3(a), 3(b), and 3(c). The samples are projected onto new variables, called principal components. Since these projections have been normalized, values in the plot reflect how similar each sample is to a given principal component. The PLS performed on the absorbance spectra discriminates the excipients well. Figure 3(a) reveals the PC plot of ethanol. Even though all the excipients are distributed well, most of them lie closer and form a group, which shows higher similarity in the samples used for calibration. Out of the first three principal components (PC), contribution of the first PC is the highest (69 percent). 17 percent and 13 percent are the contributions of the second and third PCs, respectively. The contributions of the first 3 PCs for the determination of sucrose concentration are 74 percent, 11 percent, and 13 percent, respectively. Figure 3(c) shows few groups of excipients that are randomly distributed in the score plot of tartaric acid, which denotes the resemblance of samples used for calibration. Variance values for

Table 1: Calibration statistics for ethanol, sucrose, and tartaric acid.

| Analyte | PLS factors | Wavelength ($cm^{-1}$) | No. of variables | Calibration $R^2$ | Calibration RMSEC | Validation $R^2$ | Validation RMSEV |
|---|---|---|---|---|---|---|---|
| Ethanol | 5 | 900–1500 | 312 | 0.9910 | 0.2043 | 0.9896 | 0.2193 |
| Sucrose | 5 | 900–1500 | 312 | 0.9962 | 0.1062 | 0.9956 | 0.1142 |
| Tartaric acid | 4 | 900–1500 | 312 | 0.9898 | 0.1656 | 0.9888 | 0.1699 |

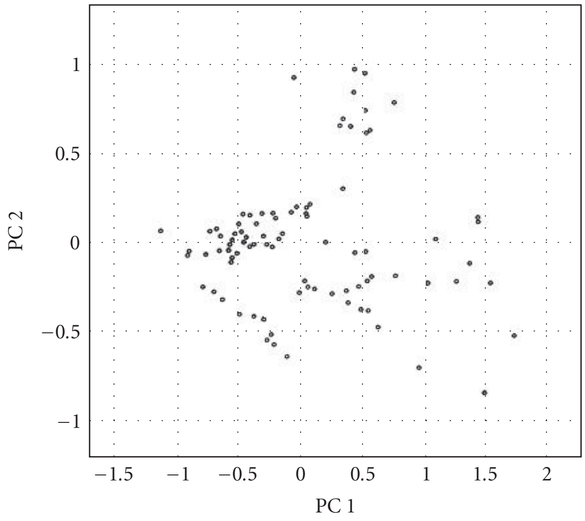

(a) Score plot of PC1 versus PC2 for ethanol in 900–1500 $cm^{-1}$ region

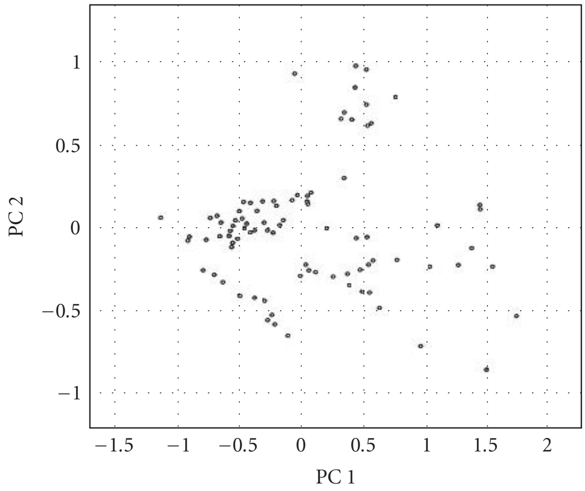

(b) Score plot of PC1 versus PC2 for sucrose in 900–1500 $cm^{-1}$ region

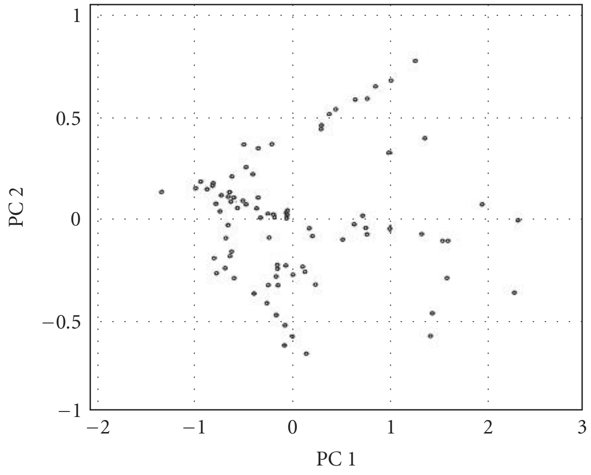

(c) Score plot of PC1 versus PC2 for tartaric acid in 900–1500 $cm^{-1}$ region

Figure 3

tartaric acid through the 3 PCs are 69 percent, 21 percent, and 8 percent, respectively.

The concentration residual is a parameter that gives an idea about the range of variation that the predicted values of concentration exhibit for the samples involved in calibration procedure. Figure 4(a) represents the concentration residual of ethanol for factor 5. It may be observed that the residual ranges from + 0.7 to − 0.4, where most of the samples lie in the region ± 0.4. Moreover, majority of the residual values lie close to zero. Figures 4(b) and 4(c) show the residual values

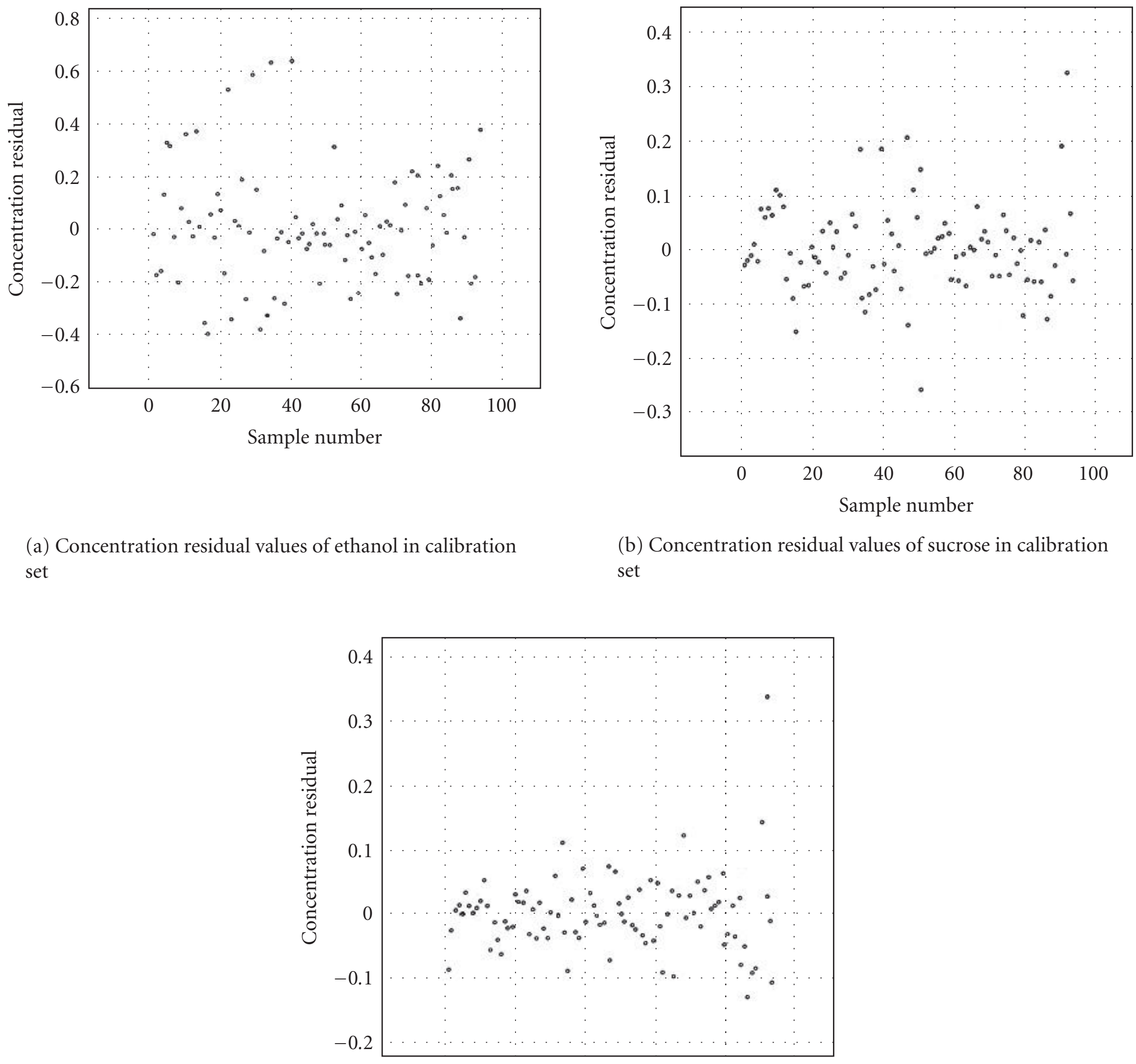

(a) Concentration residual values of ethanol in calibration set

(b) Concentration residual values of sucrose in calibration set

(c) Concentration residual values of tartaric acid in calibration set

FIGURE 4

of sucrose and tartaric acid for their optimum factors 5 and 4, respectively. It is vivid that most of the concentration residual values for both the components lie within the range of ± 0.2.

Six commercially available alcoholic beverages branded Brihans Brandy, Red Riband Vodka, Blue Riband Gin, Old-Monk Rum, McDowell's Whisky, and Royal Visa Red wine were procured from market for analysis. The calibration model obtained for ethanol was then used to quantify ethanol in real samples. The comparison of results pertaining ethanol concentration between reference analysis and the ATR technique gave satisfactory results. Figure 5 shows the accuracy of results predicted through ATR. It is vivid from Figure 5 that the explored ATR technique may very well be an effective alternate to chemical analysis.

### 4. CONCLUSION

In this paper, ATR technique was investigated as an alternate to chemical analysis. A successful calibration model with cross validation was developed with one hundred synthetic samples that yielded good correlation coefficient ($R^2$) values 0.9910, 0.9962, 0.9898 and lower RMSEC values 0.2043, 0.1062, 0.1656 for ethanol, sucrose, and tartaric acid, respectively. Comparison between ATR results to that of chemical analysis of ethanol gave promising agreement in case of real samples. Hence it may be concluded that the ATR technique may be good and effective alternate for chemical analysis for quantifying constituents in alcoholic beverages. ATR method is fast, accurate, and easy to handle for determination of the constituents in alcoholic beverages.

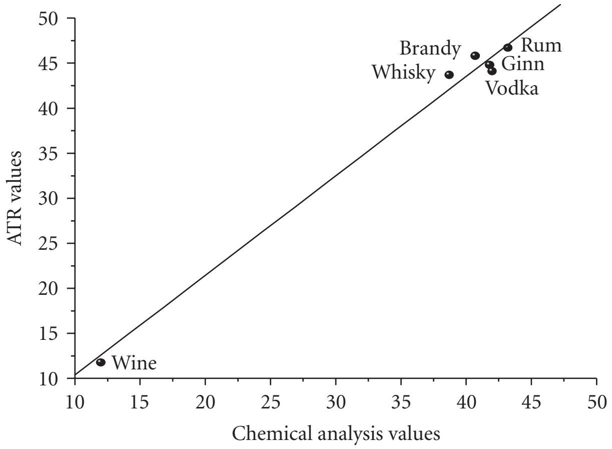

Figure 5: Predicted values versus reference values of ethanol (in percentage).

### 5. ACKNOWLEDGMENT

One of the authors, R. Nagarajan, is thankful to the Council of Scientific and Industrial Research (CSIR), India, for its support in pursuing the research work.

### REFERENCES

[1] A. Edelmann, J. Diewok, K. C. Schuster, and B. Lendl, "Rapid method for the discrimination of red wine cultivars based on mid-infrared spectroscopy of phenolic wine extracts," *Journal of Agricultural and Food Chemistry*, vol. 49, no. 3, pp. 1139–1145, 2001.

[2] J. W. Gardner and P. N. Bartlett, *Electronic Noses, Principles and Applications*, Oxford University Press, Oxford, UK, 1999.

[3] I. Herberle, A. Liebminger, U. Weimer, and W. Gopel, "Optimised sensor arrays with chromatographic preseparation: characterisation of alcoholic beverages," *Sensors and Actuators B: Chemical*, vol. 68, no. 1–3, pp. 53–57, 2000.

[4] R. Vonach, B. Lendl, and R. Kellner, "High-performance liquid chromatography with real-time Fourier-transform infrared detection for the determination of carbohydrates, alcohols and organic acids in wines," *Journal of Chromatography. A*, vol. 824, no. 2, pp. 159–167, 1998.

[5] H. Laber, J. Schulz, W. R. Sponholz, and W. Bremser, "Use of a combined GC/FTIR/MS system for the analysis of spirits," *Fresenius' Journal of Analytical Chemistry*, vol. 351, no. 6, pp. 530–535, 1995.

[6] A. W. Jones, "Measuring ethanol in saliva with QED enzymatic test device: comparison of results with blood and breath alcohol concentration," *Journal of Analytical Toxicology*, vol. 19, pp. 169–174, 1995.

[7] L. P. McClosky and L. L. Replogle, "Evaluation of an enzymatic method for estimating ethanol in wines using an "Enzyme Kit"," *American Journal of Enology and Viticulture*, vol. 25, no. 4, pp. 194–197, 1974.

[8] P. J. Worsfold, J. Růžička, and E. H. Hansen, "Rapid automated enzymatic method for the determination of alcohol in blood and beverages using flow injection analysis," *The Analyst*, vol. 106, pp. 1309–1317, 1981.

[9] A. G. Cavinato, D. M. Mayes, Z. Ge, and J. B. Callis, "Noninvasive method for monitoring ethanol in fermentation processes using fiber-optic near-infrared spectroscopy," *Analytical Chemistry*, vol. 62, no. 18, pp. 1997–1982, 1990.

[10] E. D. Dumoulin, B. P. Azais, and J. T. Guerain, "Determination of sugar and ethanol content in aqueous products of molasses distilleries by near infrared spectrometry," *Journal of Food Science*, vol. 52, pp. 626–630, 1987.

[11] M. Gallignani, S. Garrigues, and M. de la Guardia, "Direct determination of ethanol in all types of alcoholic beverages by near-infrared derivative spectrometry," *The Analyst*, vol. 118, pp. 1167–1173, 1993.

[12] M. Gallignani, S. Garrigues, and M. de la Guardia, "Derivative Fourier transform infrared spectrometric determination of ethanol in alcoholic beverages," *Analytica Chimica Acta*, vol. 287, no. 3, pp. 275–283, 1994.

[13] J. Herrera, A. Guesalaga, and E. Agosin, "Shortwave near infrared spectroscopy for non-destructive determination of maturity of wine grapes," *Measurement Science and Technology*, vol. 14, no. 5, pp. 689–697, 2003.

[14] C.-D. Patz, A. David, K. T. Hente, et al., "Wine analysis using FTIR," *Vitic. Enol. Sci.*, vol. 54, pp. 80–84, 1999.

[15] V. Bellon, "Fermentation control using ATR and an FT-IR spectrometer," *Sensors and Actuators B: Chemical*, vol. 12, no. 1, pp. 57–64, 1993.

[16] D. Picque, D. Lefier, R. Grappin, and G. Corrieu, "Monitoring of fermentation by infrared spectrometry: alcoholic and lactic fermentation," *Analytica Chimica Acta*, vol. 279, no. 1, pp. 67–72, 1993.

[17] M. Meurens and S. M. Yan, *Hand Book of Vibrational Spectroscopy*, vol. 5, John Wiley & Sons, New York, NY, USA, 2001.

[18] Indian Standard: Methods of test for alcoholic drinks, IS: 3752-1967.

[19] H. F. Shurvell, *Handbook of Vibrational Spectroscopy*, vol. 1, John Wiley & Sons, New York, NY, USA, 2001.

